# Decreased global myocardial perfusion at adenosine stress as a potential new biomarker for microvascular disease in systemic sclerosis: a magnetic resonance study

**DOI:** 10.1186/s12872-018-0756-x

**Published:** 2018-01-30

**Authors:** Tom Gyllenhammar, Mikael Kanski, Henrik Engblom, Dirk M. Wuttge, Marcus Carlsson, Roger Hesselstrand, Håkan Arheden

**Affiliations:** 1 Skane University Hospital, Department of Clinical Physiology, Lund University, Lund, Sweden; 2 Skane University Hospital, Department of Rheumatology, Lund University, Lund, Sweden

**Keywords:** Systemic sclerosis, Scleroderma, Coronary sinus flow, Cardiovascular magnetic resonance imaging, Microvascular disease

## Abstract

**Background:**

Patients with systemic sclerosis (SSc) have high cardiovascular mortality even though there is no or little increase in prevalence of epicardial coronary stenosis. First-pass perfusion on cardiovascular magnetic resonance (CMR) have detected perfusion defects indicative of microvascular disease, but the quantitative extent of hypoperfusion is not known. Therefore, we aimed to determine if patients with SSc have lower global myocardial perfusion (MP) at rest or during adenosine stress, compared to healthy controls, quantified with CMR.

**Methods:**

Nineteen SSc patients (17 females, 61 ± 10 years) and 22 controls (10 females, 62 ± 11 years) underwent CMR. Twelve patients had limited cutaneous SSc and 7 patients had diffuse cutaneous SSc. One patient had pulmonary arterial hypertension (PAH). MP was quantified using coronary sinus flow (CSF) measurements at rest and during adenosine stress, divided by left ventricular mass (LVM).

**Results:**

There was no difference in MP at rest between patients and controls (1.1 ± 0.5 vs. 1.1 ± 0.3 ml/min/g, *P* = 0.85) whereas SSc patients showed statistically significantly lower MP during adenosine stress (3.1 ± 0.9 vs. 4.2 ± 1.3 ml/min/g, *P* = 0.008). Three out of the 19 SSc patients showed fibrosis in the right ventricle insertion points despite absence of PAH. None had signs of myocardial infarction.

**Conclusions:**

Patients with SSc have decreased MP during adenosine stress compared to healthy controls. Thus hypoperfusion at stress may be a sensitive marker of cardiac disease in SSc patients possibly signifying microvascular myocardial disease.

## Background

Systemic sclerosis (SSc) is an autoimmune disease commonly recognized by its variety of multisystem autoimmune activation, fibrosis, and microvascular disease which yields a high cardiopulmonary mortality [1]. Macrovascular disease is present in many autoimmune inflammatory diseases such as rheumatoid arthritis and systemic lupus erythematosus [2]. SSc patients show high prevalence of cardiovascular mortality [1, 3–5] even with no or little epicardial stenosis [6–8]. This is likely explained by impaired myocardial microcirculation which may lead to repeated focal ischemia, myocardial fibrosis, and diastolic dysfunction [9].

First-pass perfusion on cardiovascular magnetic resonance (CMR) has demonstrated high prevalence of myocardial perfusion (MP) defects in SSc patients with no previous cardiac event [10, 11]. First-pass perfusion during adenosine hyperemia is a well-established method to detect relative MP defects but cannot quantify total left ventricular (LV) MP [12] which is necessary to assess the global microvascular status of the left ventricle.

Since the majority of the perfusion of the LV myocardium drains into the right atrium through the coronary sinus, measurement of the coronary sinus flow (CSF) reveals the global LV MP both at rest and adenosine hyperemia [13]. This can be quantified with CMR as ml/min/g myocardium [14–19].

The aim of this study was to investigate if the global MP in SSc patients is decreased at rest and during adenosine stress compared to healthy controls.

## Methods

### Study population

The study was approved by the regional ethics committee, Lund, Sweden. Written informed consent was obtained from the subjects. Consecutive patients fulfilling the inclusion criteria of being diagnosed with SSc in compliance with the American College of Rheumatology/European League against Rheumatism 2013 classification criteria for systemic sclerosis [20] were prospectively asked to participate in the study. Patients with claustrophobia or suspected hypersensitivity to adenosine were excluded. Healthy volunteers within the same age range were included in the study.

### Cardiovascular magnetic resonance

Cardiovascular magnetic resonance (CMR) was performed on a 1.5 T MR scanner with a 32-channel coil; either a Philips Achieva (Philips Healthcare, Best, the Netherlands) or a Siemens Magnetom Aera (Siemens Healthcare GmbH, Erlangen, Germany). Left ventricular (LV) function and mass was obtained from short-axis cine imaging covering the LV acquired with a steady-state free precession (SSFP) sequence during end-expiratory breath-hold.

**Fig. 1:**
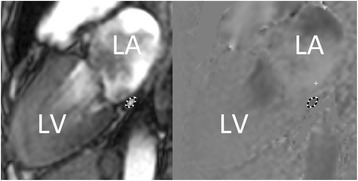
Typical example of coronary sinus delineation (dashed line) in a patient with systemic sclerosis (SSc). Left: Anatomical image; Right: Corresponding phase-contrast image. LA = Left atrium, LV = left ventricle

The coronary sinus flow (CSF) image plane was planned with guidance from a basal LV short-axis image as shown in Fig. 1. The image plane was defined to obtain a cross section of the coronary sinus as close as possible to the orifice in the right atrium to include as many LV cardiac veins as possible. The middle cardiac vein was included to measure the total flow of the whole LV. CSF was measured at rest and after 5 min of adenosine (Life Medical, Stockholm, Sweden) infusion (140 μg/kg/min) using phase-encoded breath-hold turbo field echo (TFE) velocity mapping sequence as previously described with a spatial resolution of (1.7–2.1) × (1.9–2.1) × (8-10 mm) and a VENC of 80 cm/s (rest) or 120–150 cm/s (stress) [14, 17, 18].

Late gadolinium enhancement (LGE) imaging was performed with a 3D–inversion recovery gradient-echo (IR GRE) sequence acquired during mid-diastole during end-expiratory breath-hold. Short-axis slices covering the entire LV and three long-axis projections were collected 10–20 min after an intravenous administration of additional 0.1 mmol/kg of Dotarem (Guirbet, Roissy, France) after the resting perfusion. CMR sequence parameters are listed in Table 1.

**Table 1:** CMR sequence parameters

Sequence parameters	Coronary sinus flow		Cine imaging		LGE	
	Philips	Siemens	Philips	Siemens	Philips	Siemens
Repetition time [ms]	5	5	2.9	2.5	4.2	8.3
Echo Time [ms]	2.6	2.8	1.5	1.1	1.3	3.2
Flip angle [degrees]	15	20	60	69	15	25
Inversion/saturation time [ms]	n/a	n/a	n/a	n/a	220–280	300
segmentation factor	4	4	17	17		20
Aquired spatial res. [mm]	2.1 × 2.1 × 10	1.7 × 1.9 × 8	2 × 2 × 8	2.2 × 2.2 × 8	1.5 × 1.5 × 8	1.3 × 1.8 × 8
Reconstructed spatial res. [mm]	1.1 × 1.1 × 10	1.6 × 1.6 × 8	1.3 × 1.3 × 8	2.2 × 2.2 × 8	1.5 × 1.5 × 8	1.3 × 1.3 × 8
Aquired temporal res. [ms]	34	40	50	40	n/a	n/a
Reconstructed time phases	20	20	30	25	n/a	n/a
SENSE/GRAPPA factor	2	2	2	2	0	2
VENC [cm/s]	80–150	80–120	n/a	n/a	n/a	n/a
Number of slices/heartbeat	1	1	n/a	n/a	n/a	n/a
Slice gap [mm]	0	0	0	0	0	1.6
Slice thickness [mm]	10	8	8	8	8	8

*LGE* late gadolinium enhancement, *SENSE* sensitivity encoding, *GRAPPA* generalized autocalibrating partial parallel acquisition, *VENC* velocity encoding

### CMR image analysis

Images were analyzed using the image analysis software Segment version 2.0 R5014 [21, 22]. The coronary sinus was manually delineated in the phase images (Fig. 1, right panel) over the entire cardiac cycle to measure the total CSF (mL/min) of one cardiac cycle. The corresponding magnitude images were used for guidance to localize anatomical structures (Fig. 1, left panel). In four of the controls, the coronary sinus flow could not accurately be measured at rest due to a gracile coronary sinus. However, during adenosine hyperemia, the coronary sinus flow was successfully obtained in these controls.

LV mass (LVM) was obtained from manual delineations of the endocardium and epicardium of the LV in the short-axis SSFP cine images at both end-systole and end-diastole. LVM was calculated as the myocardial volume multiplied by the myocardial density (1.05 g/mL). MP (ml/min/g) was quantified as CSF/LVM (ml/min/g).

**Fig. 2:**
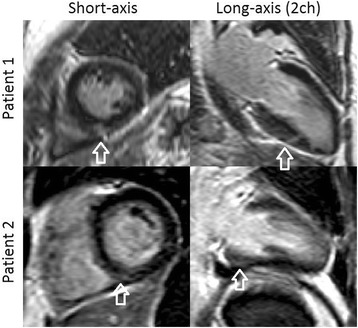
Typical example of right ventricular insertion point fibrosis marked with arrows in two patients with systemic sclerosis (SSc)

The degree of fibrosis was visually graded in LGE images by an experienced investigator (HE) (Fig. 2).

### Statistics

Statistical analyses were performed using (IBM SPSS Statistics for Windows, Version 23.0). Results are presented as mean ± SD. Because of the small cohort size, the Mann-Whitney non-parametric test was used to compare groups. Results with *P* < 0.05 were considered statistically significant.

## Results

The study included 19 SSc patients (17 females, 61 ± 10 years, range 45–74 years) and 22 controls (10 females, 62 ± 11 years, range 44–84 years). Medical history was retrieved from the patient records and none had previous history of ischemic heart disease.

The participants’ characteristics are shown in Table 2. The hemodynamic response during adenosine is shown in Table 3.

**Table 2:** Patient characteristics

		Controls	SSc patients	*P*
Age [years]		62 ± 11	61 ± 10	0.824
Sex [n]	female	10	17	
	male	12	2	
BMI [kg/m^2^]		25 ± 3	26 ± 5	0.714
LVM/BSA [g/m^2^]		58 ± 11	54 ± 12	0.089
EDV/BSA [ml/m^2^]		84 ± 12	76 ± 12	0.036*
ESV/BSA [ml]		32 ± 8	27 ± 7	0.069
CI [l/min/m^2^]		3.2 ± 0.4	3.6 ± 0.6	0.014
Skin involvement [n]	diffuse		7	
	limited		12	
Disease duration [years]			10 ± 6	
Antibody [n]	ANA		6	
	ACA		5	
	ARA		5	
	ATA		6	
PAH [n]			1	
VC % expected			96 ± 12	
DL_CO_ % expected			80 ± 19	
VC % predicted / DL_CO_ % predicted			1.3 ± 0.3	
Uric acid [μmol/L]			262 ± 59	
Telangiectasias [n]			14	
Pitting scars [n]			5	
Nailfold capillary density [loops/mm]			5.0 ± 1.3	
Skin score < 10 [n]			18	
Skin score 10–20 [n]			1	
Skin score > 20 [n]			1	
Nifedipine [n]			12	
ERA [n]			2	
PDE5I [n]			5	

Continues variables are presented as mean ± SD. * *P* < 0.05

*BSA* body surface area, *BMI* Body mass index, *LVM* left ventricular mass, *LV EDV* left ventricular end diastolic volume, *LV ESV* left ventricular end systolic volume, *CI* cardiac index, *PAH* pulmonary arterial hypertension, *VC* vital capacity, *DLCO* diffusing capacity of the lung for carbon monoxide, *ANA* anti nuclear antibodies other than ACA, ARA or ATA, *ACA* anti centromeric antibodies, *ARA* anti RNA polymerase III antibodies, *ATA* anti topoisomerase I antibodies, *ERA* endothelin receptor antagonist, *PDE5I* phosphodiesterase type 5 inhibitor

**Table 3:** Hemodynamic response during adenosine vs. rest

	Controls	SSc patients	*P*
Rest			
HR [bpm]	65 ± 10	73 ± 11	0.026*
Systolic BP [mmHg]	124 ± 13	127 ± 20	0.78
Diastolic BP [mmHg]	73 ± 9	71 ± 13	0.55
MP [ml/min/g]	1.1 ± 0.3†	1.1 ± 0.5	0.85
Adenosine stress			
HR [bpm]	85 ± 15	93 ± 13	0.05
Systolic BP [mmHg]	124 ± 17	124 ± 19	0.70
Diastolic BP [mmHg]	70 ± 11	65 ± 10	0.23
MP [ml/min/g]	4.2 ± 1.3	3.1 ± 0.9	0.008*
CFR	4.3 ± 1.1†	3.5 ± 1.9	0.09

Variables are presented as mean ± SD. * *P* < 0.05. † *N* = 18

*HR* heart rate, *BP* blood pressure, *MP* myocardial perfusion, *CFR* coronary sinus flow reserve (MP stress / MP rest)

### Global myocardial perfusion by coronary sinus flow

**Fig. 3:**
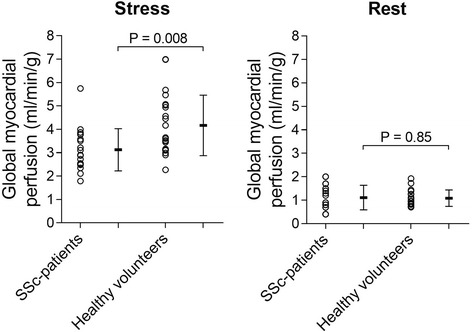
Global myocardial perfusion at rest and during adenosine stress for patients with SSc and healthy volunteers. Note the statistically significant difference in myocardial perfusion during stress. The error bars show mean ± SD

At rest there was no significant difference in MP between patients and controls (1.1 ± 0.5 vs. 1.1 ± 0.3 ml/min/g, *P* = 0.85). During adenosine infusion, however, SSc patients showed a significantly lower MP compared to controls (3.1 ± 0.9 vs. 4.2 ± 1.3 ml/min/g, *P* = 0.008, Fig. 3). MP did not differ between diffuse cutaneous and limited cutaneous SSc at rest (0.9 ± 0.1 vs. 1.2 ± 0.2 ml/min/g, *P* = 0.23), or adenosine stress (3.0 ± 0.5 vs. 3.1 ± 0.2 ml/min/g, *P* = 0.26). Two SSc patients had a substantially higher skin score than the rest of the SSc group and concurrently the lowest MP at stress in the same group (patient_1_ skin score = 14, stress MP = 1.8 ml/min/g, and patient_2_ skin score = 49, stress MP = 2.1 ml/min/g). The nailfold capillary density was 5.0 ± 1.3 loops/mm and there was no correlation between nailfold capillary density and MP at stress (*p* = 0.92).

### Fibrosis by late gadolinium enhancement

Late gadolinium enhancement (LGE) images were not obtained in three SSc patients because of inability to withstand further scanning due to discomfort. Three out of the nineteen SSc patients examined with LGE had septal fibrosis in the right ventricular insertion points. None of these patients had signs of previous myocardial infarction.

## Discussion

To the best of our knowledge this study is the first to demonstrate and quantify that patients with SSc have lower global MP during stress compared to healthy controls. Lower global MP was found even in patients with rather mild disease suggesting that disturbed MP occurs irrespective of degree of fibrotic skin involvement.

Atherosclerosis in the large coronary vessels is not more frequent in patients with SSc compared to controls, but atherosclerosis in the arterioles is [23]. Regional ischemia caused by epicardial atherosclerosis is possible to detect with CMR first pass perfusion or single-photon emission computed tomography (SPECT) [12, 24]. However, to detect diffuse global microvascular impairment requires different techniques. Positron emission tomography can quantify MP and detect microvascular disease but is associated with radiation exposure from the radioactive tracers used. To overcome these limitations, coronary sinus flow (CSF) measurements by cardiovascular magnetic resonance (CMR) was used in this study to offer precise quantification of MP without ionizing radiation [14, 15, 17].

A sub-group of patients with specific concern would be those with both microvascular and epicardial disease where the epicardial involvement needs intervention. Therefore, once microvascular disease is detected, the patients might be referred for adenosine-stress CMR or adenosine-stress SPECT to confirm or exclude localized ischemia as a sign of epicardial disease. Recently, a fully quantitative MR first-pass perfusion sequence was developed that can detect both epicardial coronary as well as microvascular disease [25]. Patients who reveal significant localized subendocardial hypoperfusion within a coronary artery supply area, should be considered for coronary angiography.

In 1985, Kahan et al. showed that the coronary flow reserve in patients with diffuse cutaneous SSc is significantly reduced during dipyridamole induced stress using invasive measurements during catheterization. Our results are in agreement with this work and also show that the coronary flow reserve (CFR) is reduced in patients with the limited cutaneous form [26]. This is in line with Montisci et al. who showed decreased coronary flow reserve measured in the left anterior descending coronary artery using transthoracic Doppler in both limited and diffuse cutaneous SSc [27]. These results were confirmed by Vacca et al. who performed myocardial multi-detector computed tomography to rule out the influence of possible epicardial stenosis [28]. However, unlike CMR, echocardiography lacks the possibility to quantify perfusion which means that a patient with a balanced decrease of rest and stress MP could present with normal coronary flow reserve. The possibility to quantify global MP in absolute numbers at both rest and stress overcomes the limitations of relative measurements of perfusion reserve. This gives extended information when analyzing perfusion abnormalities.

In our study there was no difference in global MP at rest between SSc patients and the control group. This may indicate that there are no MP abnormalities at rest. In our study the majority of SSc patients were treated with calcium channel blockers. The observation of equal MP at rest in SSc patients and controls may hypothetically be a treatment effect of calcium channel blockers increasing MP at rest from a decreased level to normal. In a study by Vignaux et al. [29], segmental perfusion defects at rest that did not correspond to any specific epicardial coronary distribution area demonstrated using first-pass perfusion. The perfusion defects in these segments where improved after two weeks of calcium-channel blocker treatment suggesting a microvascular cause of the MP abnormalities in these patients.

Primary cardiac involvement in SSc patients has high clinical importance, and it has even been suggested that primary cardiac involvement as a cause of death is underestimated because it is obscured by the secondary cardiac changes due to pulmonary hypertension [5, 30, 31]. SSc patients with perfusion defects visible on SPECT possess a high risk of developing symptomatic heart disease or death of any cause compared to patients with few or no segments with perfusion defects on SPECT [32]. As demonstrated in our study, CSF measurements using CMR is a sensitive method not only capable of detecting relative perfusion between rest and stress, but also of quantifying it in these two physiological states. Future studies should be considered to evaluate the clinical significance of such practice.

We found fibrosis in the right ventricle insertion points in three out of the nineteen investigated patients. We believe that this fibrosis is more likely a result of mechanical stress from a pre-clinical hypertensive pulmonary circulation, than an effect of local microvascular dysfunction since the MP at stress was at level with the other patients within the SSc group (stress MP = 3.6, 3.8 and 2.5 ml/min/g). To find out if microvascular function might be the cause of fibrosis in the insertion points, the study design would require the demonstration of localized hypoperfusion in the insertions points before the appearance of fibrosis.

A proposed chain of histopathological changes in which microvascular dysfunction causes fibrotic replacement throughout the body is referred to as “the loss of angiogenesis” [33]. Endothelial cell injury triggers downstream capillary enlargement leading to microhemorrhages. The pro-angiogenic system attempt to repair the vessels but fibrotic changes becomes prominent over time [33]. Furthermore, this hypothesis is supported by a recent murine model study showing prominent fibrotic changes in the small and medium sized vessels through a potent self-driving autocrine loop with transforming growth factor-β [34]. It has been suggested that microvascular impairment characterized by repeated focal ischemia lead to myocardial fibrosis in SSc patients [35, 36]. In a recent study by Barison et al., [37] myocardial extracellular volume (ECV) was shown to be increased in SSc patients without heart failure. The increased ECV was present even in patients with no myocardial fibrosis on late gadolinium enhancement (LGE) images. This supports the hypothesis that microvascular dysfunction precede other pathophysiological changes of the myocardium in SSc patients.Both peripheral microvascular remodeling, i.e. decreased capillary density [38], and uric acid levels [39] correlate with disease severity. In our study, however, neither nailfold capillary density nor uric acid levels correlated with disease severity (Table 2).

### Limitations

Though the prognostic value of global MP would be of great interest, it’s not accessible from this cross-sectional study. We did not perform first-pass perfusion imaging in this study. The combination of first-pass perfusion and coronary sinus flow (CSF) measurements could be of high interest to examine if decreased global MP (MP) is present in the absence of segmental perfusion defects. Due to low number of males in the SSc group, gender stratification was not possible.

Our study did not investigate if environmental factors correlates with microvascular dysfunction in SSc. Recent studies support the hypothesis that the epigenetic influence from environmental factors such as silica dust, drugs or infectious agents, may be important risk factors for SSc [40, 41]. Furthermore, the limited size of this study does not enable sufficient power for correlation analyses between MP and the clinical features of the patients.

## Conclusions

Patients with systemic sclerosis have lower global myocardial perfusion during adenosine stress compared to healthy controls. Thus, hypoperfusion at stress may be a sensitive marker of cardiac disease in patients with systemic sclerosis and may signify microvascular disease.

Therefore, systemic sclerosis patients in which cardiac microvascular dysfunction is suspected may be considered for CMR. However, studies showing the prognostic impact of hypoperfusion in these patients and the effect of therapeutic regimes on myocardial perfusion are needed.

## Abbreviations

CMR: Cardiovascular magnetic resonance
CSF: Coronary sinus flow
IR GRE: 3D–inversion recovery gradient-echo
LGE: Late gadolinium enhancement
LV: Left ventricular
LVM: LV mass
MP: Myocardial perfusion
SSc: Systemic sclerosis
SSFP: Steady-state free precession
TFE: Phase-encoded breath-hold turbo field echo

## References

[1] Tyndall AJ, Bannert B, Vonk M, Airo P, Cozzi F, Carreira PE (2010). Causes and risk factors for death in systemic sclerosis: a study from the EULAR scleroderma trials and research (EUSTAR) database. Ann Rheum Dis.

[2] Shoenfeld Y (2005). Accelerated atherosclerosis in autoimmune rheumatic diseases. Circulation.

[3] Steen VD, Medsger TA (2000). Severe organ involvement in systemic sclerosis with diffuse scleroderma. Arthritis Rheum.

[4] Czirják L, Kumánovics G, Varjú C, Nagy Z, Pákozdi a SZ (2008). Survival and causes of death in 366 Hungarian patients with systemic sclerosis. Ann Rheum Dis.

[5] Elhai M, Meune C, Avouac J, Kahan A, Allanore Y. Trends In mortality in patients with systemic sclerosis over 40 years: a systematic review and meta-analysis of cohort studies. Rheumatology (Oxford) 2012;51 September 2011:1017–1026. 10.1093/rheumatology/ker269 21900368

[6] Akram MR, Handler CE, Williams M, Carulli MT, Andron M, Black CM (2006). Angiographically proven coronary artery disease in scleroderma. Rheumatology.

[7] Au K, Singh MK, Bodukam V, Bae S, Maranian P, Ogawa R (2011). Atherosclerosis in systemic sclerosis: a systematic review and meta-analysis. Arthritis Rheum.

[8] Cannarile F, Valentini V, Mirabelli G, Alunno A, Terenzi R, Luccioli F (2015). Cardiovascular disease in systemic sclerosis. Ann Transl Med.

[9] Allanore Y, Meune C, Kahan A (2008). Systemic sclerosis and cardiac dysfunction: evolving concepts and diagnostic methodologies. Curr Opin Rheumatol.

[10] Mavrogeni SI, Bratis K, Karabela G, Spiliotis G, Van wijk K, Hautemann D (2015). Cardiovascular magnetic resonance imaging clarifies cardiac pathophysiology in early, asymptomatic diffuse systemic sclerosis. Inflamm Allergy - Drug Targets.

[11] Rodriguez-Reyna TS, Morelos-Guzman M, Hernandez-Reyes P, Montero-Duarte K, Martinez-Reyes C, Reyes-Utrera C, et al. Assessment of myocardial fibrosis and microvascular damage in systemic sclerosis by magnetic resonance imaging and coronary angiotomography. Rheumatology. 2014; 10.1093/rheumatology/keu350 25239881

[12] Jerosch-Herold M (2010). Quantification of myocardial perfusion by cardiovascular magnetic resonance. J Cardiovasc Magn Reson.

[13] Hood WB (1968). Regional venous drainage of the human heart. Br Heart J.

[14] Schwitter J, DeMarco T, Kneifel S, von Schulthess GK, Jorg MC, Arheden H (2000). Magnetic resonance-based assessment of global coronary flow and flow reserve and its relation to left ventricular functional parameters : a comparison with positron emission tomography. Circulation.

[15] Lund GK, Wendland MF, Shimakawa A, Arheden H, Ståhlberg F, Higgins CB (2000). Coronary sinus flow measurement by means of velocity-encoded cine MR imaging: validation by using flow probes in dogs. Radiology.

[16] Arheden H, Saeed M, Törnqvist E, Lund G, Wendland MF, Higgins CB (2001). Accuracy of segmented MR velocity mapping to measure small vessel pulsatile flow in a phantom simulating cardiac motion. J Magn Reson Imaging.

[17] Bloch KM, Carlsson M, Arheden H, Ståhlberg F (2009). Quantifying coronary sinus flow and global LV perfusion at 3T. BMC Med Imaging.

[18] Carlsson M, Jögi J, Markenroth Bloch K, Hedén B, Ekelund U, Ståhlberg F, et al. Submaximal adenosine induced coronary hyperemia with 12 hours caffein abstinence; implications for clinical adenosine perfusion imaging tests. Clin Physiol Funct Imaging. 2013 10.1111/cpf.12125 24418159

[19] Gyllenhammar T, Fernlund E, Jablonowski R, Jögi J, Engblom H, Liuba P, et al. Young patients with hypertrophic cardiomyopathy, but not subjects at risk, show decreased myocardial perfusion reserve quantified with CMR. Eur Heart J Cardiovasc Imaging. 2014; 10.1093/ehjci/jeu137 25139907

[20] van den Hoogen F, Khanna D, Fransen J, Johnson SR, Baron M, Tyndall A (2013). 2013 classification criteria for systemic sclerosis: an American college of rheumatology/European league against rheumatism collaborative initiative. Ann Rheum Dis.

[21] Heiberg E, Sjögren J, Ugander M, Carlsson M, Engblom H, Arheden H. Design and validation of segment - freely available software for cardiovascular image analysis. BMC Med Imaging. 2010;10(1) 10.1186/1471-2342-10-1 PMC2822815 20064248

[22] Segment version 2.0 R5014. http://segment.heiberg.se. Accessed 1 Jan 2018.

[23] D’Angelo WA, Fries JF, AT M, Shulman LE (1969). Pathologic observations in systemic sclerosis (scleroderma). Am J Med.

[24] Underwood SR, Anagnostopoulos C, Cerqueira M, Ell PJ, Flint EJ, Harbinson M (2004). Myocardial perfusion scintigraphy: the evidence. Eur J Nucl Med Mol Imaging.

[25] Engblom H, Xue H, Akil S, Carlsson M, Hindorf C, Oddstig J (2017). Fully quantitative cardiovascular magnetic resonance myocardial perfusion ready for clinical use: a comparison between cardiovascular magnetic resonance imaging and positron emission tomography. J Cardiovasc Magn Reson.

[26] Kahan A, Nitenberg A, Foult J-M, Amor B, Menkes C-J, Devaux J-Y (1985). Decreased coronary reserve in primary scleroderma myocardial disease. Arthritis Rheum.

[27] Montisci R, Vacca A, Garau P, Colonna P, Ruscazio M, Passiu G (2003). Detection of early impairment of coronary flow reserve in patients with systemic sclerosis. Ann Rheum Dis.

[28] Vacca A, Montisci R, Garau P, Siotto P, Piga M, Cauli A (2013). Prognostic impact of coronary microcirculation abnormalities in systemic sclerosis: a prospective study to evaluate the role of non-invasive tests. Arthritis Res Ther..

[29] Vignaux O, Allanore Y, Meune C, Pascal O, Duboc D, Weber S (2005). Evaluation of the effect of nifedipine upon myocardial perfusion and contractility using cardiac magnetic resonance imaging and tissue Doppler echocardiography in systemic sclerosis. Ann Rheum Dis.

[30] Meune C, Avouac J, Wahbi K, Cabanes L, Wipff J, Mouthon L (2008). Cardiac involvement in systemic sclerosis assessed by tissue-doppler echocardiography during routine care: a controlled study of 100 consecutive patients. Arthritis Rheum.

[31] Hesselstrand R, Scheja a A a (1998). Mortality and causes of death in a Swedish series of systemic sclerosis patients. Ann Rheum Dis.

[32] Steen VD, Follansbee WP, Conte CG, T a M (1996). Thallium perfusion defects predict subsequent cardiac dysfunction in patients with systemic sclerosis. Arthritis Rheum.

[33] Matucci-Cerinic M, Manetti M, Bruni C, Chora I, Bellando-Randone S, Lepri G (2017). The “myth” of loss of angiogenesis in systemic sclerosis: a pivotal early pathogenetic process or just a late unavoidable event?. Arthritis Res Ther.

[34] Wermuth PJ, Carney KR, Mendoza FA, Piera-Velazquez S, Jimenez SA. Endothelial cell-specific activation of transforming growth factor-β signaling in mice induces cutaneous, visceral, and microvascular fibrosis. Lab Investig. 2017; 10.1038/labinvest.2017.23 PMC6530474 28346399

[35] Kahan A, Allanore Y. Primary myocardial involvement in systemic sclerosis. Rheumatology. 2006;45(SUPPL. 4) 10.1093/rheumatology/kel312 16980717

[36] Meune C, Vignaux O, Kahan A, Allanore Y (2010). Heart involvement in systemic sclerosis: evolving concept and diagnostic methodologies. Arch Cardiovasc Dis.

[37] Barison A, Gargani L, De Marchi D, Aquaro GD, Guiducci S, Picano E (2015). Early myocardial and skeletal muscle interstitial remodelling in systemic sclerosis: insights from extracellular volume quantification using cardiovascular magnetic resonance. Eur Heart J Cardiovasc Imaging.

[38] Corrado A, Correale M, Mansueto N, Monaco I, Carriero A, Mele A, et al. Nailfold capillaroscopic changes in patients with idiopathic pulmonary arterial hypertension and systemic sclerosis-related pulmonary arterial hypertension. 2017. 10.1016/j.mvr.2017.06.005 28619664

[39] Gigante A, Barbano B, Barilaro G, Quarta S, Gasperini ML, Di Mario F, et al. Serum uric acid as a marker of microvascular damage in systemic sclerosis patients. Microvasc Res. 2016; 10.1016/j.mvr.2016.03.007 27003713

[40] Sato Saigusa S, Ichimura Y, Takahashi T, Toyama T, Yoshizaki A, Taniguchi T (2017). vasculopathy in systemic sclerosis endothelial cells, contributing to the development of fibrosis and Fli1 deficiency induces CXCL6 expression in dermal fibroblasts and Fli1 deficiency induces CXCL6 expression in dermal fibroblasts and endothelial cells. J Rheumatol.

[41] Epigenetic AY. Suppression of Fli1, a potential predisposing factor in the pathogenesis of systemic sclerosis. Int J Biochem Cell Biol. 2015; 10.1016/j.biocel.2015.06.004 26055516

